# The world Polleniidae (Diptera, Oestroidea): key to genera and checklist of species

**DOI:** 10.3897/zookeys.971.51283

**Published:** 2020-09-29

**Authors:** Silvia Gisondi, Knut Rognes, Davide Badano, Thomas Pape, Pierfilippo Cerretti

**Affiliations:** 1 Department of Biology and Biotechnologies ‘Charles Darwin’, Sapienza University of Rome, Piazzale A. Moro 5, 00185, Rome, Italy; 2 Natural History Museum of Denmark, Universitetsparken 15, 2100 Copenhagen, Denmark; 3 Faculty of Arts and Education, Department of Early Childhood Education, University of Stavanger, NO-4036 Stavanger, Norway; 4 DISTAV, University of Genoa, Corso Europa 26, 16132, Genoa, Italy; 5 Australian National Insect Collection, CSIRO National Facilities and Collections, Black Mountain, Canberra, Australia

**Keywords:** Calliphoridae, Calyptratae, catalogue, cluster flies, key, parasitoids, taxonomy

## Abstract

A key to the world genera and a checklist of the world species for the family Polleniidae, including distributions, are provided. The following taxonomic and nomenclatural changes are proposed: *Nitellia hermoniella* Lehrer, 2007 = *Pollenia mediterranea* Grunin, 1966, **syn. nov.**, *Pollenia bentalia* Lehrer, 2007 = *Pollenia semicinerea* Villeneuve, 1911, **syn. nov.**, *Dasypoda angustifrons* Jacentkovský, 1941 = *Pollenia tenuiforceps* Séguy, 1928, **syn. nov.**; *Anthracomyza* Malloch, 1928, **resurrected name** (monotypic; type species *Anthracomyia atratula* Malloch) is considered a valid name and tentatively assigned to Polleniidae, giving *Anthracomyza atratula* (Malloch, 1927) as a **resurrected combination**; *Morinia crassitarsis* (Villeneuve, 1936), **stat. rev.** is considered a valid species, and *Micronitellia* Enderlein, 1936, **stat. nov.** is considered an available name.

## Introduction

The family Polleniidae (cluster flies) is a small group of oestroid flies comprising 147 species (Cerretti et al. 2019 and present paper). The family group name was originally proposed by Brauer and Bergenstamm (1889) to include the single genus *Pollenia* Robineau-Desvoidy. Later, the Old World *Polleniasensu lato* (i.e., including the morphologically diverging New Zealand *Pollenia* species), the Oriental genera *Dexopollenia* Townsend and *Xanthotryxus* Aldrich, and the Nearctic genus *Melanodexia* Williston were treated in Calliphoridae as composing the subfamily Polleniinae (or tribe Polleniini) (e.g., Hall 1965; Dear 1986; Schumann 1986; Kurahashi 1989). The group was then re-circumscribed by Rognes (1991a) to include *Morinia* Robineau-Desvoidy, *Nesodexia* Villeneuve and (tentatively) *Wilhelmina* Villeneuve, the latter being reassigned to the calliphorid subfamily Phumosiinae by Rognes (2011), which is followed here. More recently, Cerretti et al. (2019) elevated the group to full family rank and gave morphological and molecular evidence to support both the monophyly of the Polleniidae and the inclusion of six taxa previously assigned to the Rhinophoridae, namely *Alvamaja chlorometallica* Rognes and five Afrotropical species moved from the genus *Phyto* Robineau-Desvoidy into *Morinia* (Cerretti et al. 2020).

During the last few years molecular data consistently retrieved the Polleniidae (almost always represented only by *Pollenia*) as sister to the Tachinidae and phylogenetically distant from the ‘core’ Calliphoridae (e.g., Singh and Wells 2013; Winkler et al. 2015; Cerretti et al. 2017; Blaschke et al. 2018; Kutty et al. 2019; Stireman et al. 2019) but this sister group relationship has remained practically without support from morphological evidence: the currently most convincing non-molecular synapomorphy could well be the breeding habit as parasitoids of soil-dwelling invertebrates. Tachinids are parasitoids of other arthropods, and groups near the base of the family develop on soil-dwelling insect larvae (Cerretti et al. 2014; Stireman et al. 2019); the natural history and host range information of polleniids is limited to a handful of *Pollenia* species (Keilin 1915; Tawfik and El-Husseini 1971; Yahnke and George 1972; Rognes 1991a; Szpila 2003; Grzywacz et al. 2012; El Husseini 2019), and all of these develop as endoparasitoids in earthworms. Marshall (2020) observed a native New Zealand *Pollenia* displaying what can only be interpreted as oviposition behaviour, extending the ovipositor into a mixture of loose soil and organic debris. Recent field observations of adults of an unidentified species of *Melanodexia* revealed that females have a similar behaviour to that observed for several *Pollenia* and other parasitoids of soil-dwelling organisms: they can be seen walking frenetically on bark lying on the ground, keeping wings folded on their back (SG pers. obs. 2019, California).

**Figure 1.**
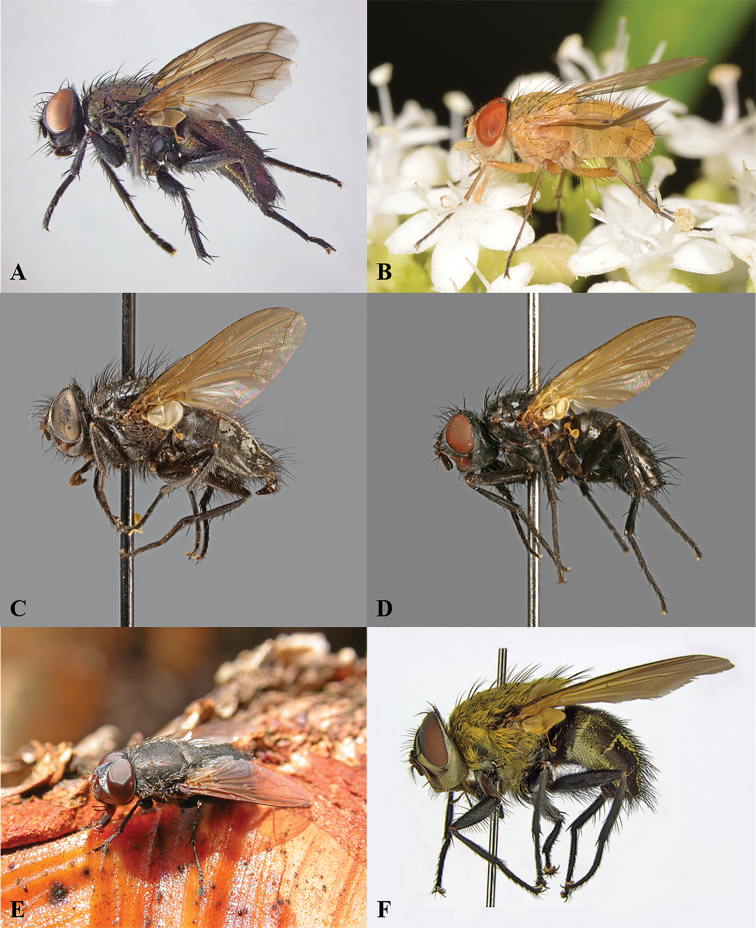
Representative species for all genera of Polleniidae. **A***Alvamaja chlorometallica* Rognes [Romania] **B**Dexopollenia cf. flava (Aldrich) [Japan: Honshu] **C***Melanodexia satanica* Shannon [USA: California] **D***Morinia tsitsikamma* Cerretti et al. [South Africa] **E***Pollenia* sp. [Italy] **F***Xanthotryxus mongol* Aldrich [China]. Photographs: Cerretti et al. (2019) (**A**), K. Oomori (**B**), P. Cerretti (**E**), K. Szpila (**F**).

**Figure 2.**
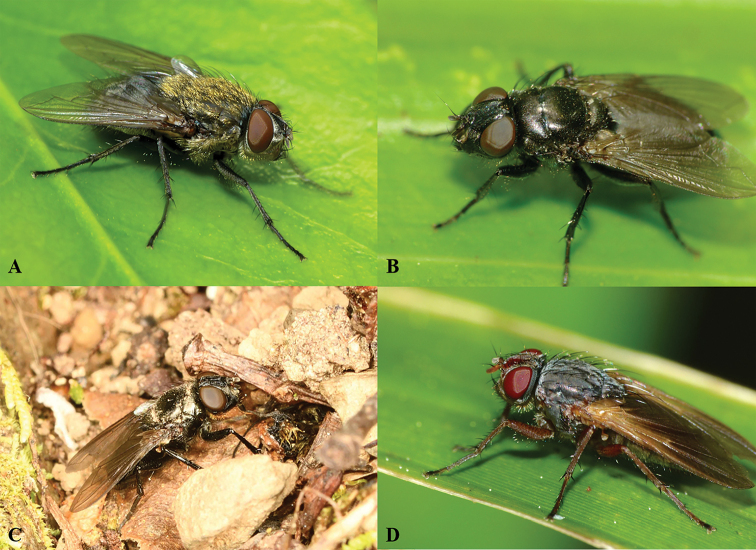
Diversity of the genus *Pollenia*. **A***Pollenia pediculata* Macquart [New Zealand] **B***Pollenia pernix* (Hutton) [New Zealand] **C**Pollenia nr. pernix [New Zealand] **D***Pollenia uniseta* Dear [New Zealand]. Photographs: S. Kerr (**A, B, D**), S. Marshall (**C**).

We recognise 147 species of Polleniidae classified into eight genera (Fig. 1). *Pollenia* is the most species-rich and widespread genus, with 95 species described from the Palaearctic, Oriental and Australasian regions [and seven species known from the Nearctic Region as introductions (Rognes 1991a; Whitworth 2006; Jewiss-Gaines et al. 2012, Bowser 2015)] (Fig. 2). The remaining seven genera are considerably less diverse: the single species assigned to *Alvamaja* Rognes (*A. chlorometallica* Rognes) is recorded from a few localities in southeastern Europe, *Dexopollenia* comprises 21 species from the southeastern Palaearctic and the Oriental Region, the Nearctic endemic genus *Melanodexia* includes eight species, *Morinia* contains 13 species from the Afrotropical and Palaearctic regions and *Xanthotryxus* includes seven species from south-eastern Palaearctic and the Oriental Region. The remaining genera, the monotypic Australian *Anthracomyza* Malloch and the Palaearctic *Nesodexia* Villeneuve, are here tentatively assigned to the family. Remarkably, there are no Polleniidae recorded from the Neotropical Region, neither native nor introduced.

Many regional catalogues and keys to polleniid genera (and species) have been published in the recent decades (e.g., Hall 1948; James 1955, 1970, 1977; Lehrer 1972; Pont 1980; Shewell 1987; Rognes 1991a, 1998; Kurahashi 1995; Jewiss-Gaines et al. 2012; Pape et al. 2015), and an incomplete key to genera is available from Peris (2004). The aim of the present paper is to lay the foundation for future taxonomic and phylogenetic studies by producing a key to the world genera of the family Polleniidae and a checklist of the world species.

## Materials and methods

### Key to genera

A dichotomous key to the adults of both sexes was constructed to contain the genera here considered members of the Polleniidae. This means that we are excluding the monotypic genera *Wilhelmina* Schmitz & Villeneuve, 1932 and *Nepenthomyia* Kurahashi & Beaver, 1979, both associated with pitcher plants of the genus *Nepenthes* and not considered polleniids. *Wilhelmina* was considered a possible member of Polleniidae (or Polleniinae, or Polleniini) by Schmitz and Villeneuve (1932), Fan (1965, 1992, 1997) and Rognes (1991a), but we follow Rognes (2011) in suggesting its reassignment to Phumosiinae. *Nepenthomyia* was considered “closely related” to *Wilhelmina* by Kurahashi and Beaver (1979), although with no indication of subfamily assignment, and a position within the Polleniinae was not accepted by Rognes (1991a). The key was constructed through direct examination of available material and from literature data. No specimens of *Anthracomyza* were examined and characters for it were derived from the original description by Malloch (1927).

Digital images of the specimens shown in Fig. 1C, D were taken using a Canon EOS 6D camera equipped with Canon Photo lens MP-E 65 mm 1.2.8 and processed by Canon Digital Photo Professional (Canon: Ōta, Tokyo, Japan), Combine ZM by Alan Hadley and GIMP 2.10.4 by Alexandre Prokoudine.

### World checklist

The world checklist is based on original literature, though following the papers by Rognes (1987b, 1991a, 1998, 2010), Evenhuis et al. (2004, 2010, 2015, 2016), O’Hara et al. (2011), Cerretti et al. (2019) and literature therein. It lists all currently valid nominal genera and species of the family Polleniidae including their synonyms and associated *nomina nuda*. Incorrect subsequent spellings have been entered to the extent they have come to our knowledge. Valid names of taxa are arranged alphabetically according to genus and species (no subfamilial or tribal classification is recognised here). Each genus-group name is listed with author, year, page, type species with author and date, and form of type fixation with author and date. Each type species is given in its original binomen (ICZN 1999; recommendation 67B), and if that name is a junior synonym it is followed by the valid name of the species in square brackets. Each species-group name is listed with author, year, page, type locality in standardised modern spelling (original quote in parenthesis if needed to avoid confusion), and relevant nomenclatural details (homonymy, lectotype designations, etc.). Unavailable names are listed with an explanation as to their unavailability, and incorrect subsequent spellings are given with the relevant reference. New specific synonyms are based on comparisons of the original descriptions of the nominal species in question with material (specimens, photos, illustrations) available to the authors. Additional information may be given under “Remarks”. Distributional data are based on the literature and online databases (Fauna Europaea [Pape et al. 2015] for Palaearctic species) but we do not refer all records to their original sources. Records have been entered to the extent they have come to our knowledge and they are reported for countries and major islands, except that larger countries are recorded at the level of state or province.

### Caveat for key users

Notwithstanding strong molecular evidence suggesting the monophyly of Polleniidae, members of the group apparently do not share any unique morphological apomorphy. For this reason, it is not possible to provide a simple and clear-cut diagnosis of this oestroid family since several exceptions have to be taken into account once given a common set of characters. Therefore, we refer to Cerretti et al.’s (2019) diagnosis of the Polleniidae, though highlighting all the uncertainties related to it.

**Diagnosis.** Small to medium-sized oestroid flies varying from yellow to black in ground colour. Facial sclerite at least weakly carinate [with few exceptions, e.g. *Pollenia griseotomentosa* (Jacentkovský)]. Stem vein bare dorsally. Anal vein not reaching wing margin. Posterodorsal margin of hind coxa bare. Prosternum and proepisternal depression bare. Postalar wall setose (occasionally bare in small specimens of *Morinia*). Female: ovipositor sclerite length moderate; sternite 8 of ovipositor elongate with posterior margin entire; cerci long and narrow. Male: ventral and ventrolateral surface of distalmost parts of distiphallus smooth.

A comprehensive phylogeny including all the eight genera recognised as cluster flies is still awaiting, as well as a thorough revision of the family, therefore generic boundaries within this family are still labile due to the absence of molecular evidence and strong morphological characters. For these reasons, we are here applying a traditional generic division without proposing any subfamilial or tribal classification nor any new generic synonyms. We anticipate future rearrangements, such as *Dexopollenia* synonymised with *Pollenia*, or the exclusion of *Nesodexia*. Moreover, the New Zealand *Pollenia* are still entirely untouched in a phylogenetic context.

## Results

### Key to genera

**Table:** 

1	Simultaneously: body ground colour entirely or largely yellow or testaceous; thorax (occasionally also abdomen) with sparse golden crinkly hair-like setae; parafacial bare below anteriormost frontal seta; subcostal sclerite with only a few pale setulae or without setulae among micropubescence; lower calypter broad	***Dexopollenia* Townsend [in part**]
–	Body ground colour prevalently black, sometimes with metallic reflections; if abdomen largely yellow, then parafacial bare. Other combination of characters	**2**
2	Simultaneously: body ground colour black with metallic green or bronze-violet reflections; parafacial bare; thorax without sparse golden crinkly hair-like setae; lower calypter narrow, tongue-shaped; anterior and posterior fringes of metathoracic spiracle subequal in size; node at base of R_4+5_ with 1–3 fine setulae; slender bodied flies	***Alvamaja* [*chlorometallica*] Rognes**
–	Body colouration without metallic reflections; if body black with bronze, green, blue or violet metallic reflections then other combination of characters	**3**
3	Thorax with numerous golden crinkly hair-like setae in addition to ground setulae	**4**
–	Thorax without golden crinkly hair-like setae	**7**
4	Simultaneously: scutum usually with 1 + 2 intra-alar setae [0 + 2 in some New Zealand species]; hind tibia with posterodorsal preapical seta not differentiated. Parafacial setulose on nearly whole length. Body ground colour varying from metallic or non-metallic black [Palaearctic species] to metallic green/blue or violet [New Zealand species], rarely abdomen yellow [*P. bicolor* Robineau-Desvoidy]. Lower calypter broad. Subcostal sclerite usually with a bundle of long black or yellow setae among the micropubescence [usually absent in New Zealand species]	***Pollenia* Robineau-Desvoidy [in part**]
–	Simultaneously: scutum with 0 + 2 intra-alar setae; hind tibia with anterodorsal, dorsal and posterodorsal preapical setae subequal in size. Parafacial setulose or bare. Body colouration not metallic. Lower calypter broad or narrow. Subcostal sclerite with or without a bundle of long black or yellow setae among the micropubescence	**5**
5	Parafacial entirely setulose	***Pollenia* Robineau-Desvoidy [in part, Australia**]
–	Parafacial bare or with at most a few setulae in the upper half [Palaearctic and Oriental]	**6**
6	Parafacial bare except for the extreme uppermost part where a few short setulae are usually present below the anteriormost frontal seta. One or 2 presutural acrostichal setae. Two to 4 postpronotal setae. Subcostal sclerite with numerous black or yellow setulae among the micropubescence. Coxopleural streak absent. Lappets of metathoracic spiracle dark brown. Large-sized species [except for *X. ludigensis* Fan]	***Xanthotryxus* Aldrich**
–	Parafacial entirely bare below anteriormost frontal seta. One presutural acrostichal seta. Two postpronotal setae. Subcostal sclerite bare or at most with a few pale setulae among micropubescence. Coxopleural streak absent or present. Lappets of metathoracic spiracle yellow or dark. Small to medium-sized species	***Dexopollenia* Townsend [in part**]
7	Node at base of R_4+5_ setulose dorsally	**8**
–	Node at base of R_4+5_ bare dorsally. [Lower calypter narrow. Scutum with 0 + 2 intra-alar setae. Hind tibia with 3 preapical setae (i.e., anterodorsal, dorsal and posterodorsal), all approximately the same size]	**9**
8	Parafacial bare. Scutum with 1 + 3 intra-alar setae and 4 postsutural acrostichal setae. Large, robust species with white microtomentose stripes on thorax and a chequered abdomen	***Nesodexia* Villeneuve [tentatively assigned to Polleniidae, see below**]
–	Parafacial setulose. Scutum with 0–1 + 2 intra-alar setae, 2 or 3 postsutural acrostichal setae. Thorax and abdomen shiny black. Small to medium sized species	***Anthracomyza* Malloch [tentatively assigned to Polleniidae, see below**]
9	Two marginal scutellar setae [Old World]	***Morinia* Robineau-Desvoidy**
–	Three to 5 marginal scutellar setae [New World]	***Melanodexia* Williston**

### World checklist

#### Family Polleniidae Brauer & Bergenstamm, 1889

Polleniidae Brauer & Bergenstamm, 1889: 85 (17). Type genus *Pollenia* Robineau-Desvoidy, 1830. Without description or definition, but available “by an indication” i.e., by being formed before 1931 “from an available generic name” (ICZN 1999; articles 12.1 and 12.2.4).

Moriniini Townsend, 1919: 546. Type genus *Morinia* Robineau-Desvoidy, 1830.

Melanodexiini Hall, 1948: 351. Type genus *Melanodexia* Williston, 1893.

#### Genus *Alvamaja* Rognes, 2010

*Alvamaja* Rognes, 2010: 4. Type species: *Alvamaja chlorometallica* Rognes, 2010, by original designation.

***Alvamaja chlorometallica* Rognes, 2010**

*Alvamaja chlorometallica* Rognes, 2010: 4. Type locality: Serbia, Pčinja District, Vranjska Banja.

**Distribution.** Palaearctic – Romania, Serbia.

#### Genus *Dexopollenia* Townsend, 1917

*Dexopollenia* Townsend, 1917: 201. Type species: *Dexopollenia testacea* Townsend, 1917, by original designation.

***Dexopollenia aurantifulva* Feng, 2004**

*Dexopollenia aurantifulva* Feng, 2004: 806. Type locality: China, Sichuan, Ya’an, Mt. Zhougong, 1760 m.

**Distribution.** Palaearctic – China (Sichuan).

***Dexopollenia bicolor* Malloch, 1935**

*Dexopollenia bicolor* Malloch, 1935: 671. Type locality: Malaysia, Perak, Bukit Larut (Larut Hills).

*Pollenia mallochi* Blackith, 1991: 271. Unnecessary new replacement name for *Dexopollenia bicolor* Malloch, 1935.

**Distribution.** Oriental – Malaysia (West Malaysia), Thailand.

***Dexopollenia bicoloripes* Malloch, 1931**

*Dexopollenia bicoloripes* Malloch, 1931: 199. Type locality: Malaysia, Selangor, Bukit Kutu.

**Distribution.** Oriental – Malaysia (West Malaysia).

***Dexopollenia chrysothrix* Bezzi, 1927**

*Dexopollenia chrysothrix* Bezzi, 1927: 231. Type locality: Australia, New South Wales, Kiuskin [sic] (locality not found).

**Distribution.** Australasian – Australia (New South Wales).

***Dexopollenia disemura* Fan & Deng, 1993**

*Dexopollenia disemura* Fan & Deng in Fan, Feng & Deng, 1993: 201. Type locality: China, Sichuan, Mt. Emei, Jinding.

**Distribution.** Palaearctic – China (Sichuan).

***Dexopollenia fangensis* Kurahashi, 1995**

*Dexopollenia fangensis* Kurahashi, 1995: 141. Type locality: Thailand, Fang, Doi Huai Hwer, 1231 m.

**Distribution.** Oriental – Thailand, Vietnam.

***Dexopollenia flava* (Aldrich, 1930)**

*Lispoparea flava* Aldrich, 1930: 5. Type locality: China, Sichuan, Mt. Emei.

**Distribution.** Oriental – India, Taiwan. Palaearctic – China (Sichuan), Japan (Honshu).

***Dexopollenia geniculata* Malloch, 1935**

*Dexopollenia geniculata* Malloch, 1935: 671. Type locality: China, Sichuan, Mt. Emei.

**Distribution.** Oriental – China (Yunnan), Laos. Palaearctic – China (Sichuan).

***Dexopollenia hirtiventris* Malloch, 1935**

*Dexopollenia hirtiventris* Malloch, 1935: 669. Type locality: Malaysia, Pahang, Bukit Fraser (= Fraser’s Hill).

**Distribution.** Oriental – Malaysia (West Malaysia).

***Dexopollenia luteola* (Villeneuve, 1927)**

*Pollenia luteola* Villeneuve, 1927: 393. Type locality: Taiwan, Kosempo and Taihorinsho.

**Distribution.** Oriental – Taiwan.

***Dexopollenia maculata* (Villeneuve, 1933)**

*Lispoparea maculata* Villeneuve, 1933b: 196. Type locality: China, Sichuan, Mt. Emei.

**Distribution.** Oriental – Taiwan. Palaearctic – China (Sichuan).

***Dexopollenia monsdulitae* (Senior-White, Aubertin & Smart, 1940)**

*Pollenia monsdulitae* Senior-White, Aubertin & Smart, 1940: 131. Type locality: Malaysia, Sarawak, Mt. Dulit, 1219 m.

**Distribution.** Oriental – Malaysia (Sabah, Sarawak).

***Dexopollenia nigra* Kurahashi, 1987**

*Dexopollenia nigra* Kurahashi, 1987: 66. Type locality: Papua New Guinea, Southern Highlands, Margarima, Walk River.

**Distribution.** Australasian – Papua New Guinea.

***Dexopollenia nigriscens* Fan, 1992**

*Dexopollenia nigriscens* Fan, 1992: 530. Type locality: China, Xizang, Bomi, Yegong, 3050 m.

**Distribution.** Oriental – Nepal. Palaearctic – China (Xizang).

***Dexopollenia papua* Kurahashi, 1987**

*Dexopollenia papua* Kurahashi, 1987: 64. Type locality: Papua New Guinea, Southern Highlands, Margarima (“Margarima Farm”), 2000 m.

**Distribution.** Australasian – Papua New Guinea.

***Dexopollenia sakulasi* Kurahashi,1987**

*Dexopollenia sakulasi* Kurahashi, 1987: 68. Type locality: Papua New Guinea, Sandaun Province (= West Sepik Province), Torricelli Mts, 900 m.

**Distribution.** Australasian – Papua New Guinea.

***Dexopollenia testacea* Townsend, 1917**

*Dexopollenia testacea* Townsend, 1917: 201. Type locality: India, Assam, Mangaldai District, Assam-Bhutan Frontier, Jany [sic] (locality not found).

**Distribution.** Oriental – India, Nepal.

***Dexopollenia tianmushanensis* Fan, 1997**

*Dexopollenia tianmushanensis* Fan in Fan (Ed.), 1997: 430. Type locality: China, Zhejiang, Mt. Tianmushan.

**Distribution.** Palaearctic – China (Zhejiang).

***Dexopollenia trifascia* (Walker, 1861)**

*Musca trifascia* Walker, 1861: 245. Type locality: Indonesia, Western New Guinea (= Irian Jaya), Dorey.

**Distribution.** Oriental – Indonesia (Western New Guinea).

***Dexopollenia uniseta* Fan, 1992**

*Dexopollenia uniseta* Fan in Fan (Ed.), 1992: 529. Type locality: China, Xizang, Cuona.

*Dexopollenia wyatti* Kurahashi, 1992: 24. Type locality: Malaysia, Sabah, Mt. Kinabalu, Lumu Lumu, 152 m.

**Distribution.** Oriental – Malaysia (Sabah). Palaearctic – China (Xizang).

***Dexopollenia yuphae* Kurahashi, 1995**

*Dexopollenia yuphae* Kurahashi, 1995: 140. Type locality: Thailand, Kanchana Buri, near Sai Yok.

**Distribution.** Oriental – Laos, Thailand, Vietnam.

#### Genus *Melanodexia* Williston, 1893

*Melanodexia* Williston, 1893: 256. Type species: *Melanodexia tristis* Williston, 1893, by monotypy.

*Melanodexiopsis* Hall, 1948: 351. Type species: *Melanodexiopsis tristina* Hall, 1948, by original designation.

*Mellanodexmia*: Sidhu et al. (2018: 22). Incorrect subsequent spelling of *Melanodexia* Williston, 1893.

***Melanodexia californica* Hall, 1948**

*Melanodexia californica* Hall, 1948: 354. Type locality: USA, California, Placerville.

**Distribution.** Nearctic – USA (California).

***Melanodexia glabricula* (Bigot, 1887)**

*Nitellia glabricula* Bigot, 1887: clxxiv. Type locality: USA, California.

**Distribution.** Nearctic – USA (California).

***Melanodexia grandis* (Shannon, 1926)**

*Melanodexiopsis grandis* Shannon, 1926: 138. Type locality: USA, California, Monterey County.

*Melanodexiopsis pacifica* Hall, 1948: 359. Type locality: USA, California, Monterey County, Pacific Grove.

**Distribution.** Nearctic – USA (California).

***Melanodexia idahoensis* (Hall, 1948)**

*Melanodexiopsis idahoensis* Hall, 1948: 357. Type locality: USA, Idaho, Genesee.

**Distribution.** Nearctic – USA (Idaho).

***Melanodexia nox* (Hall, 1948)**

*Melanodexiopsis nox* Hall, 1948: 358. Type locality: USA, Oregon, Hood River.

**Distribution.** Nearctic – USA (California, Oregon, Washington).

***Melanodexia satanica* Shannon, 1926**

*Melanodexia satanica* Shannon, 1926: 138. Type locality: USA, California, Fresno County, Los Gatos Canyon.

**Distribution.** Nearctic – USA (California, Washington).

***Melanodexia tristina* (Hall, 1948)**

*Melanodexiopsis tristina* Hall, 1948: 359. Type locality: USA, California, San Bernardino County.

**Distribution.** Nearctic – USA (California, Colorado).

***Melanodexia tristis* Williston, 1893**

*Melanodexia tristis* Williston, 1893: 257. Type locality: USA, California, “southern California” and Monterey County.

**Distribution.** Nearctic – USA (California).

#### Genus *Morinia* Robineau-Desvoidy, 1830

*Morinia* Robineau-Desvoidy, 1830: 264. Type species: *Morinia velox* Robineau-Desvoidy, 1830 [= *Musca doronici* Scopoli, 1763], by subsequent designation (Rondani, 1862: 159).

*Calobatemyia* Macquart, 1855b: 33. Type species: *Calobatemyia nigra* Macquart, 1855b [= *Musca doronici* Scopoli, 1763], by original designation.

*Anthracomya* Rondani, 1856: 87. Type species: *Anthracomya geneji* Rondani, 1856 [= *Musca doronici* Scopoli, 1763], by original designation.

*Morjnia* Rondani, 1862: 151. Unjustified emendation of *Morinia* Robineau-Desvoidy, 1830, *teste*O’Hara et al. (2011).

*Antracomya* Lioy, 1864: 881. Unjustified emendation of *Anthracomya* Rondani, 1856.

*Anthracomyia* Rondani, 1868: 50. Unjustified emendation of *Anthracomya* Rondani, 1856.

*Disticheria* Enderlein, 1934: 188. *Nomen nudum*. [Type species given as *Musca melanoptera* Fallén, 1817, but no description.]

*Anthromyia*: Sidhu et al. (2018: 22). Incorrect subsequent spelling of *Anthracomya* Rondani, 1856.

***Morinia argenticincta* (Senior-White, 1923)**

*Idiopsis argenticincta* Senior-White, 1923: 48. Type locality: India, Himachal Pradesh, Shimla.

**Distribution.** Oriental – India, Nepal. Palaearctic – Japan (Honshu)

***Morinia carinata* (Pape, 1987)**

*Phyto carinata* Pape, 1987: 378. Type locality: South Africa, Western Cape, Cape Point Nature Reserve.

**Distribution.** Afrotropical – South Africa.

***Morinia doronici* (Scopoli, 1763)**

*Musca doronici* Scopoli, 1763: 333. Type locality: Slovenia [as “Carniola”].

*Musca melanoptera* Fallén, 1817: 253. Type locality: Sweden, Östergötland or Västergötland. [Lectotype designated by Rognes (1991a: 211).] Junior primary homonym of *Musca melanoptera* Gmelin, 1790: 2833 [Bombyliidae].

*Morinia velox* Robineau-Desvoidy, 1830: 265. Type locality: not stated, probably France, Yonne, Saint-Sauveur-en-Puisaye.

*Morinia fuscipennis* Robineau-Desvoidy, 1830: 265. Type locality: not stated, probably France, Yonne, Saint-Sauveur-en-Puisaye.

*Anthracomya geneji* Rondani, 1856: 87 [as *Genèji*]. Type locality: Italy.

*Calobatemyia nigra* Macquart, 1855b: 34. Type locality: Switzerland.

**Distribution.** Palaearctic – Austria, Belgium, Czech Republic, Denmark, Finland, France, Germany, Greece, Hungary, Italy, Netherlands, Norway, Poland, Russia, Slovakia, Spain, Sweden, Switzerland, Ukraine.

***Morinia crassitarsis* (Villeneuve, 1936)**

*Anthracomyia crassitarsis* Villeneuve, 1936: 7. Type locality: China, Sichuan. Stat. rev. [as var. of *Anthracomyia melanoptera* (Fallén, 1817). Subspecific status according to ICZN 1999; article 45.6.4.]

**Distribution.** Palaearctic – China (Sichuan).

**Remarks.** The name-bearing type of *M. crassitarsis* has not been located, but unpublished studies (by TP) of Chinese specimens matching the original description would seem to support a status for this nominal species as valid.

***Morinia lactineala* (Pape, 1997)**

*Phyto lactineala* Pape, 1997: 160. Type locality: South Africa, Western Cape, 10 km S Citrusdal, Koornlandskloof.

**Distribution.** Afrotropical – South Africa.

***Morinia longirostris* (Crosskey, 1977)**

*Phyto longirostris* Crosskey, 1977: 44. Type locality: South Africa, Western Cape, Cape Town, Table Mountain, slopes above cable house.

**Distribution.** Afrotropical – South Africa.

***Morinia nigerrima* (Herting, 1961)**

*Anthracomyia nigerrima* Herting, 1961: 9. Type locality: Japan, Hoshi-Gunma [sic] (likely Gunma prefecture, locality not found).

*Anthromyia nigrerrima*: Sidhu et al. (2018: 22). Incorrect subsequent spelling of *Anthracomyia nigerrima* Herting, 1961.

**Distribution.** Palaearctic – Japan (?Honshu).

***Morinia piliparafacia* Fan, 1997**

*Morinia piliparafacia* Fan in Fan et al. 1997: 438. Type locality: China, Sichuan, Mt. Gongga, 2500 m.

**Distribution.** Palaearctic – China (Sichuan).

***Morinia proceripenisa* Feng, 2004**

*Morinia proceripenisa* Feng, 2004: 806. Type locality: China, Sichuan, Mt. Erlang, 2670 m.

**Distribution.** Palaearctic – China (Sichuan).

***Morinia royi* (Pape, 1997)**

*Phyto royi* Pape, 1997: 163. Type locality: South Africa, Western Cape, Overberg District, De Hoop Nature Reserve.

**Distribution.** Afrotropical – South Africa.

***Morinia skufyini* Khitsova, 1983**

*Morinia skufyini* Khitsova, 1983: 1588. Type locality: Russia, Krasnodar Krai, Caucasus Nature Reserve, Kozlinaya balka [sic] (locality not found).

**Distribution.** Palaearctic – Russia (Krasnodar).

***Morinia stuckenbergi* (Crosskey, 1977)**

*Phyto stuckenbergi* Crosskey, 1977: 44. Type locality: South Africa, Western Cape, Bredasdorp District, Arniston coastal dunes.

**Distribution.** Afrotropical – South Africa.

***Morinia tsitsikamma* Cerretti, Stireman, Badano, Gisondi, Rognes, Lo Giudice & Pape, 2019**

*Morinia tsitsikamma* Cerretti, Stireman, Badano, Gisondi, Rognes, Lo Giudice & Pape, 2019: 964. Type locality: South Africa, Western Cape, Bloukrans Pass.

**Distribution.** Afrotropical – South Africa.

#### Genus *Pollenia* Robineau-Desvoidy, 1830

*Pollenia* Robineau-Desvoidy, 1830: 412. Type species: *Musca rudis* Fabricius, 1794, by original designation.

*Nitellia* Robineau-Desvoidy, 1830: 417. Type species: *Musca vespillo* Fabricius, 1794, *sensu* Coquillett [misidentification, = *Musca atramentaria* Meigen, 1826 *teste*Rognes (1991a: 215)], by designation of Coquillett (1910: 576). *Remarks.* The type species has been misidentified, and we here follow ICZN 1999 (*Code* Article 70.3.2) and designate the taxonomic species actually involved in the misidentification.

*Cephysa* Robineau-Desvoidy, 1863: 655, 677. Type species: *Cephysa muscidea* Robineau-Desvoidy, 1863, by monotypy.

*Orizia* Robineau-Desvoidy, 1863: 655, 678. Type species: *Orizia conjuncta* Robineau-Desvoidy, 1863, by subsequent designation (Townsend, 1916: 8).

*Chaetopollenia* Enderlein, 1936: 211 [as *Chætopollénia*]. Type species: *Musca vespillo* Fabricius, 1794, *sensu* Enderlein [misidentification, = *Musca amentaria* Scopoli, 1763 *teste*Rognes (1991a: 218)], by monotypy. *Remarks*. The type species has been misidentified, and we here follow ICZN 1999 (*Code* Article 70.3.2) and designate the taxonomic species actually involved in the misidentification.

*Micronitellia* Enderlein, 1936: 211 [as *Micronitéllia*]. Type species: *Musca varia* Meigen, 1826, by monotypy. Stat. nov. *Remarks.* We here consider Enderlein’s (1936) type fixation for *Micronitellia* valid, therefore we do not regard Lehrer (1967) as the first reviser as previously suggested by Rognes (1991a).

*Trichopollenia* Enderlein, 1936: 211 [as *Trichopollénia*]. Type species: *Musca vagabunda* Meigen, 1826, by monotypy.

*Polleniella* Jacentkovský, 1941a: 15, 16. *Nomen nudum*. [No description.]

*Buresiella* Jacentkovský, 1941b: 21, 22 [as *Burešiella*]. Type species: *Pollenia pallida* Rodendorf, 1926, by monotypy.

*Dasypollenia* Jacentkovský, 1941b: 20, 22. *Nomen nudum*. *Remarks*. Genus-group name proposed after 1930 without designation of type species from four included species.

*Polleniella* Jacentkovský, 1941b: 20, 22. Type species: *Polleniella distincta* Jacentkovský, 1941 [= *Pollenia mayeri* Jacentkovský, 1941], by monotypy. Unavailable name; type species a *nomen nudum*. Validated by Jacentkovský (1942).

*Polleniomyia* Jacentkovský, 1941b: 20, 23. *Nomen nudum*. *Remarks*. Genus-group name proposed after 1930 without designation of type species from two included species.

*Pseudopollenia* Jacentkovský, 1941b: 21, 22. Type species: *Pollenia vera* Jacentkovský, 1936, by monotypy.

*Bureschiella* Jacentkovský, 1941c: 31. Unjustified emendation of *Buresiella* Jacentkovský, 1941. Type species: *Pollenia pallida* Rohdendorf, 1926, automatic.

*Chaetopollenia* Jacentkovský, 1941c: 31. *Nomen nudum*. [No description.]

*Dasypollenia* Jacentkovský, 1941c: 31. *Nomen nudum*. [No description. No type species designated.]

*Polleniomyia* Jacentkovský, 1941c: 31. *Nomen nudum*. [No description. No type species designated.]

*Polleniella* Jacentkovsky, 1942: 209 (17). Type species: *Pollenia mayeri* Jacentkovský, 1941a: 14.

*Dasypollenia* Jacentkovský, 1942: 210 (18). *Nomen nudum*. *Remarks*. Genus-group name proposed after 1930 without designation of type species from four included species.

*Polleniomyia* Jacentkovský, 1942: 220 (28). Type species: *Pollenia labialis* Robineau-Desvoidy, 1863, by original designation.

*Polleniomyma* Jacentkovský, 1944b: 119. Unnecessary new replacement name for *Polleniomyia* Jacentkovský, 1942.

*Eupollenia* Lehrer, 1963: 290. Type species: *Musca rudis* Fabricius, 1794, by original designation.

*Jacentkovskyiomyia* Lehrer, 1963: 292. Type species: *Polleniella griseotomentosa* Jacentkovský, 1944a, by original designation.

*Mariomyia* Lehrer, 1963: 292. Type species: *Pollenia mayeri* Jacentkovský, 1941, by original designation.

*Parapollenia* Lehrer, 1963: 290. Type species: *Pollenia dasypoda* Portschinsky, 1881, by original designation.

*Rohdendorfiomyia* Lehrer, 1963: 292. Type species: *Musca vespillo* Fabricius, 1794 *sensu* Lehrer [misidentification, = *Musca amentaria* Scopoli, 1763 *teste*Rognes (1991a: 218)], by original designation. *Remarks*. The type species has been misidentified, and we here follow ICZN 1999 (*Code* Article 70.3.2) and designate the taxonomic species actually involved in the misidentification.

*Sachtlebeniola* Lehrer, 1963: 291, 300. *Nomen nudum*. *Remarks*. Genus-group name proposed after 1930 without designation of type species from five included species.

*Seguyiomyia* Lehrer, 1963: 293 [as *Séguyiomyia*]. Type species: *Musca vagabunda* Meigen, 1826, by original designation.

*Zumptiomyia* Lehrer, 1963: 292. Type species: *Pollenia bisulca* Pandellé, 1896, by original designation.

*Dasypollenia* Lehrer, 1967: 256. Type species: *Pollenia dasypoda* Portschinsky, 1881, by original designation.

*Sepimentum* Hutton, 1901: 66. Type species: *Sepimentum fumosum* Hutton, 1901, by designation of Townsend (1916: 8).

*Huttonophasia* Curran, 1927: 354. Type species: *Gymnophania pernix* Hutton, 1901, by original designation.

***Pollenia advena* Dear, 1986**

*Pollenia advena* Dear, 1986: 32. Type locality: New Zealand, Three Kings Islands, Great Island, Castaway Camp.

**Distribution.** Australasian – New Zealand.

***Pollenia aerosa* Dear, 1986**

*Pollenia aerosa* Dear, 1986: 33. Type locality: New Zealand, South Island, Westland District, Lake Paringa.

**Distribution.** Australasian – New Zealand.

***Pollenia agneteae* Rognes, 2019**

*Pollenia agneteae* Rognes, 2019: 380. Type locality: Armenia, Aragatsotn, River Kasakh between Alagyaz and Aparan.

**Distribution.** Palaearctic – Armenia.

***Pollenia alajensis* Rohdendorf, 1926**

*Pollenia alajensis* Rohdendorf, 1926: 101 [as subspecies of *Pollenia rudis* (Fabricius, 1794)]. Type locality: Kyrgyzstan, Alayskiy Range (Alai or Alay Mts), Fergana (“Kchi Alai”) [given by Rohdendorf (1928: 338), see Rognes (1987a).]

*Pollenia sytshevskajae* Grunin, 1970: 480. Type locality: Kyrgyzstan, Terskey-Alatau Range, Chon-kyzylsu River, 2650 m.

*Pollenia sytshevskiae*: Schumann (1986: 47). Incorrect subsequent spelling of *Pollenia sytshevskajae* Grunin, 1970.

**Distribution.** Palaearctic – Kyrgyzstan.

***Pollenia amentaria* (Scopoli, 1763)**

*Musca amentaria* Scopoli, 1763. Type locality: Slovenia, road below Kranjska Gora and Tolbin. [Neotype designated by Rognes (1991a: 218).]

*Pollenia micans* Robineau-Desvoidy, 1830: 416. Type locality: not stated, probably France, Yonne, Saint-Sauveur-en-Puisaye.

*Musca nigrina* Meigen, 1838: 305. Type locality: Germany, Nordrhein-Westfalen, probably Stolberg, near Aachen [as “Hiesige Gegend”]. Junior primary homonym of *Musca nigrina* Fallén, 1817 [Tachinidae]. [Lectotype designated by Rognes (1991a: 221; as “holotype”).]

*Musca nitens* Zetterstedt, 1845: 1340. Type locality: probably Denmark. Junior primary homonym of *Musca nitens* Villers, 1789: 549 [Syrphidae]. [Lectotype designated by Rognes (1991a: 221).]

*Chaetopollenia soudeki* Jacentkovský, 1941b: 21, 22. Type locality: Czech Republic, Brno, Skolny Statek Adamov, Kanice. [Lectotype designated by Rognes (1991a: 221).]

**Distribution.** Palaearctic – Albania, Andorra, Armenia, Austria, Belgium, Bulgaria, China (Xinjiang), Croatia, Czech Republic, Denmark, Finland, France, Germany, Great Britain, Greece, Hungary, Iran, Ireland, Italy, Macedonia, Morocco, Netherlands, Norway, Poland, Romania, Russia, Slovakia, Slovenia, Spain, Sweden, Switzerland, Ukraine, Yugoslavia.

***Pollenia angustigena* Wainwright, 1940**

*Pollenia angustigena* Wainwright, 1940: 444 [as subspecies of *Pollenia rudis* (Fabricius, 1794)]. Type locality: England, Worcestershire, Abberley Hill. [Lectotype designated by Rognes (1987b: 482).]

**Distribution.** Nearctic [introduced] – Canada (British Columbia, Ontario, Quebec); USA (California, Colorado, Idaho, Maine, New Jersey, North Carolina, Ohio, Oregon, South Dakota, Utah, Virginia, Washington, Wisconsin). Oriental [introduced] – China (Guangdong). Palaearctic – Andorra, Austria, Belgium, Croatia, Czech Republic, Denmark, Finland, France, Germany, Great Britain, Hungary, Italy, Netherlands, Norway, Poland, Portugal (Madeira, mainland), Russia, Slovakia, Spain, Sweden, Switzerland, Ukraine.

***Pollenia antipodea* Dear, 1986**

*Pollenia antipodea* Dear, 1986: 34. Type locality: New Zealand, South Island, Southland District, Tiwai Point.

**Distribution.** Australasian – New Zealand.

***Pollenia astrictifrons* Dear, 1986**

*Pollenia astrictifrons* Dear, 1986: 34. Type locality: New Zealand, South Island, Nelson District, Mt. Murchison, 1350–1440 m.

**Distribution.** Australasian – New Zealand.

***Pollenia atramentaria* (Meigen, 1826)**

*Musca atramentaria* Meigen, 1826: 65. Type locality: Austria.

*Pollenia levis* Rondani, 1862: 195. Type locality: Italy, Parma or Lombardy [as “Insubria”]. [Lectotype designated by Rognes (1991c: 365).]

**Distribution.** Palaearctic – Andorra, Austria, Belarus, Czech Republic, France, Germany, Italy, Latvia, Lithuania, Netherlands, Poland, Romania, Russia, Slovakia, Spain, Switzerland, Ukraine.

***Pollenia atricoma* Dear, 1986**

*Pollenia atricoma* Dear, 1986: 34. Type locality: New Zealand, South Island, Buller District, Lewis Pass, 1050 m.

**Distribution.** Australasian – New Zealand.

***Pollenia atrifemur* Malloch, 1930**

*Pollenia atrifemur* Malloch, 1930: 321. Type locality: New Zealand, South Island, Mid Canterbury District, Upper Hororata.

**Distribution.** Australasian – New Zealand.

***Pollenia bartaki* Rognes, 2016**

*Pollenia bartaki* Rognes, 2016: 572. Type locality: Jordan, NW Ajlun, 32°19.877'N, 35°43.110'E, 850 m.

**Distribution.** Palaearctic – Jordan.

***Pollenia bezziana* Rognes, 1992**

*Pollenia bezziana* Rognes, 1992: 98. Type locality: Italy, Novara, Masera Commune, Rogna Hamlet.

**Distribution.** Palaearctic – Italy.

***Pollenia bicolor* Robineau-Desvoidy, 1830**

*Pollenia bicolor* Robineau-Desvoidy, 1830: 415. Type locality: not stated, probably France, Yonne, Saint-Sauveur-en-Puisaye.

*Pollenia guernica*: Lehrer (2007c: 21). Unavailable name; proposed without a statement that the name-bearing type will be (or is) deposited in a named collection, here listed under *Pollenia bicolor* Robineau-Desvoidy, 1830.

**Distribution.** Palaearctic – Andorra, France, Morocco, Portugal, Spain.

***Pollenia bulgarica* Jacentkovský, 1939**

*Pollenia bulgarica* Jacentkovský, 1939: 190. Type locality: Bulgaria, Sliven and Kloster Bachkovo.

**Distribution.** Palaearctic – Armenia, Azerbaijan, Bulgaria, Greece, Hungary, Iran, Moldova, Poland, Romania, Slovakia, Turkey, Ukraine.

***Pollenia calamisessa* Hardy, 1932**

*Pollenia calamisessa* Hardy, 1932: 340. Type locality: Australia, Queensland, Brisbane.

**Distribution.** Australasian – Australia (Queensland).

***Pollenia chotei* Kurahashi & Tumrasvin, 1979**

*Pollenia chotei* Kurahashi & Tumrasvin, 1979: 303. Type locality: Thailand, Nakhon Nayok Province, Khao Yai.

**Distribution.** Oriental – Thailand.

***Pollenia commensurata* Dear, 1986**

*Pollenia commensurata* Dear, 1986: 35. Type locality: New Zealand, South Island, Mid Canterbury District, Mt. Somers.

**Distribution.** Australasian – New Zealand.

***Pollenia consanguinea* Dear, 1986**

*Pollenia consanguinea* Dear, 1986: 35. Type locality: New Zealand, South Island, Central Otago District, Old Man Range, Hyde Rock, 1550–1650 m.

**Distribution.** Australasian – New Zealand.

***Pollenia consectata* Dear, 1986**

*Pollenia consectata* Dear, 1986: 35. Type locality: New Zealand, North Island, Auckland District, Huia.

**Distribution.** Australasian – New Zealand.

***Pollenia contempta* Robineau-Desvoidy, 1863**

*Pollenia contempta* Robineau-Desvoidy, 1863: 676. Type locality: France, Var, Callian. [Neotype designated by Rognes (1992: 109).]

**Distribution.** Palaearctic – France, Italy, Portugal, Spain, Tunisia.

***Pollenia cuprea* Malloch, 1930**

*Pollenia cuprea* Malloch, 1930: 323 [as var. of *demissa* Hutton, 1901]. Type locality: New Zealand, North Island, Whanganui District, Whanganui.

**Distribution.** Australasian – New Zealand.

***Pollenia dasypoda* Portschinsky, 1881**

*Pollenia dasypoda* Portschinsky, 1881: 143. Type locality: Georgia, Mtskheta.

*Dasypollenia landrocki* Jacentkovský, 1941b: 20, 22 [key]. Type locality: Czech Republic, Lednice [Eisgrub].

**Distribution.** Oriental – India, Pakistan. Palaearctic – Austria, Bulgaria, Czech Republic, Egypt, Georgia, Germany, Greece, Hungary, Iran, Israel, Italy, Kazakhstan, Lebanon, Moldova, Poland, Romania, Russia, Saudi Arabia, Slovakia, Syria, Tajikistan, Turkey, Ukraine, West Bank.

***Pollenia demissa* (Hutton, 1901)**

*Sepimentum demissa* Hutton, 1901: 67. Type locality: New Zealand, North Island, Wellington.

*Pollenia minor* Malloch, 1930: 323 [as var. of *demissa* Hutton, 1901]. Type locality: New Zealand, North Island, Whanganui District, Whanganui.

**Distribution.** Australasian – New Zealand.

***Pollenia dysaethria* Dear, 1986**

*Pollenia dysaethria* Dear, 1986: 37. Type locality: New Zealand, North Island, Auckland District Titirangi.

**Distribution.** Australasian – New Zealand.

***Pollenia dyscheres* Dear, 1986**

*Pollenia dyscheres* Dear, 1986: 37. Type locality: New Zealand, South Island, Nelson District, Mt. Owen, 1500 m.

**Distribution.** Australasian – New Zealand.

***Pollenia enetera* Dear, 1986**

*Pollenia enetera* Dear, 1986: 38. Type locality: New Zealand, South Island, Fiordland District, Fiordland National Park, Milford.

**Distribution.** Australasian – New Zealand.

***Pollenia erlangshanna* Feng, 2004**

*Pollenia erlangshanna* Feng, 2004: 803. Type locality: China, Sichuan, Mt. Erlang, 2750 m.

**Distribution.** Palaearctic – China (Sichuan).

***Pollenia eurybregma* Dear, 1986**

*Pollenia eurybregma* Dear, 1986: 38. Type locality: New Zealand, South Island, Central Otago District, Old Man Range, Hyde Rock, 1550–1650 m.

**Distribution.** Australasian – New Zealand.

***Pollenia flindersi* Hardy, 1932**

*Pollenia flindersi* Hardy, 1932: 338. Type locality: Australia, Victoria, Flinders.

**Distribution.** Australasian – Australia (Victoria).

***Pollenia fulviantenna* Dear, 1986**

*Pollenia fulviantenna* Dear, 1986: 38. Type locality: New Zealand, South Island, Buller District, Nelson Lakes National Park, west side of Lake Rotoit.

**Distribution.** Australasian – New Zealand.

***Pollenia fulvipalpis* Macquart, 1835**

*Pollenia fulvipalpis* Macquart, 1835: 270. Type locality: France, Gironde, Bordeaux.

*Pollenia bisulca* Pandellé, 1896: 152. Type locality: France, Hautes-Pyrénées, Tarbes.

*Pollenia flavipalpis*: Rondani (1862: 202). Incorrect subsequent spelling of *Pollenia fulvipalpis* Macquart, 1835.

**Distribution.** Palaearctic – Channel Islands, France, Slovakia, Spain, Switzerland.

***Pollenia fumosa* (Hutton, 1901)**

*Sepimentum fumosum* Hutton, 1901: 67. Type locality: New Zealand, South Island, “Christchurch or Ashburton”.

**Distribution.** Australasian – New Zealand.

***Pollenia griseotomentosa* (Jacentkovský, 1944)**

*Polleniella griseotomentosa* Jacentkovský, 1944a: 45. Type locality: Poland, Struga. [Neotype designated by Rognes (1991a: 225).]

**Distribution.** Nearctic [introduced] – Canada (British Columbia, Ontario); USA (New York, Western Virginia). Palaearctic – Andorra, Austria, Belarus, Belgium, Czech Republic, Denmark, Finland, France, Germany, Great Britain, Hungary, Italy (mainland, Sardinia), Latvia, Netherlands, Poland, Russia, Slovakia, Spain, Sweden, Switzerland, Turkey, Ukraine.

***Pollenia grunini* Rognes, 1988**

*Pollenia grunini* Rognes, 1988: 318. Type locality: Russia, Karachay-Cherkess Republic (Karachayevo-Cherkesskaya Respublika), 5 km S Teberda.

**Distribution.** Palaearctic – Armenia, Georgia, Russia.

***Pollenia haeretica* Séguy, 1928**

*Pollenia haeretica* Séguy, 1928: 374. Type locality: Algeria, Skikda (“Philippeville”). [Lectotype designated by Rognes (2010: 46).]

**Distribution.** Palaearctic – Algeria, Tunisia, Italy (Sardinia).

***Pollenia hazarae* (Senior-White, 1923)**

*Dexopollenia hazarae* Senior-White, 1923: 51. Type locality: Pakistan, Abbottabad, 1256 m.

**Distribution.** Oriental – India, Pakistan.

***Pollenia hirticeps* Malloch, 1927**

*Pollenia hirticeps* Malloch, 1927: 318. Type locality: Australia, New South Wales, Blue Mts.

**Distribution.** Australasian – Australia (New South Wales, South Australia).

***Pollenia hispida* Dear, 1986**

*Pollenia hispida* Dear, 1986: 39. Type locality: New Zealand, South Island, Central Otago District, Old Man Range, Hyde Rock, 1550–1650 m.

**Distribution.** Australasian – New Zealand.

***Pollenia huangshanensis* Fan & Chen, 1997**

*Pollenia huangshanensis* Fan & Chen in Fan et al. 1997: 415. Type locality: China, Anhui, Huangshan Mt., 850 m.

**Distribution.** Palaearctic – China (Anhui).

***Pollenia hungarica* Rognes, 1987**

*Pollenia hungarica* Rognes, 1987b: 483. Type locality: Hungary, Albertirsa.

**Distribution.** Palaearctic – Austria, China (Shanghai) [introduced], Czech Republic, Finland, France, Germany, Hungary, Italy, Latvia, Netherlands, Norway, Poland, Russia, Saudi Arabia, Slovakia, Sweden, Switzerland, Ukraine, Yugoslavia.

***Pollenia ibalia* Séguy, 1930**

*Pollenia ibalia* Séguy, 1930: 148. Type locality: Morocco, Moyen Atlas, Ras el Ksar, 900 m.

*Pollenia rungsi* Séguy, 1953: 88. Type locality: Morocco, Rabat.

*Pollenia funebris* Villeneuve, 1933a: 284. Type locality: Morocco, Marrakech. Junior primary homonym of *Pollenia funebris* Robineau-Desvoidy, 1863 [*nomen dubium*, *teste* Schumann, 1986].

**Distribution.** Nearctic [introduced] – Alaska. Palaearctic – Morocco.

***Pollenia immanis* Dear, 1986**

*Pollenia immanis* Dear, 1986: 40. Type locality: New Zealand, South Island, Central Otago District, Old Man Range, 1550–1650 m.

**Distribution.** Australasian – New Zealand.

***Pollenia insularis* Dear, 1986**

*Pollenia insularis* Dear, 1986: 40. Type locality: New Zealand, Stewart Island, Table Hill, 425–715 m.

**Distribution.** Australasian – New Zealand.

***Pollenia japonica* Kano & Shinonaga, 1966**

*Pollenia japonica* Kano & Shinonaga, 1966: 223. Type locality: Japan, Honshu, Miyagi Prefecture, Mt. Zao.

**Distribution.** Palaearctic – Japan (Honshu, Kyushu).

***Pollenia labialis* Robineau-Desvoidy, 1863**

*Pollenia labialis* Robineau-Desvoidy, 1863: 67. Type locality: France, Yvelines, Rambouillet. [Neotype designated by Rognes (1991a: 228).]

*Pollenia excarinata* Wainwright, 1940: 442. Type locality: United Kingdom, Wales, Tan-y-Bwlch.

**Distribution.** Nearctic [introduced] – Canada (British Columbia, Ontario, Quebec); USA (Colorado, Indiana, Maine, Michigan, New Hampshire, New Mexico, Oregon, Pennsylvania, Vermont, Washington). Palaearctic – Andorra, Austria, Belgium, Bosnia and Herzegovina, China (Anhui, Henan) [introduced], Czech Republic, Denmark, Finland, France, Germany, Great Britain, Greece, Hungary, Ireland, Italy, Latvia, Lithuania, Netherlands, Norway, Poland, Portugal, Romania, Russia, Slovakia, Spain, Sweden, Switzerland, Turkey, Ukraine.

***Pollenia lativertex* Dear, 1986**

*Pollenia lativertex* Dear, 1986: 41. Type locality: New Zealand, Stewart Island, Table Hill, 425–715 m.

**Distribution.** Australasian – New Zealand.

***Pollenia leclercqiana* (Lehrer, 1978)**

*Nitellia leclercqiana* Lehrer, 1978: 139. Type locality: Spain, Madrid, Valdemoro.

**Distribution.** Palaearctic – France, Spain (Balearic Islands, mainland), Morocco.

***Pollenia limpida* Dear, 1986**

*Pollenia limpida* Dear, 1986: 41. Type locality: New Zealand, South Island, Southland District, Mt. Barber, 1155 m.

**Distribution.** Australasian – New Zealand.

***Pollenia luteovillosa* Rognes, 1987**

*Pollenia luteovillosa* Rognes, 1987b: 490. Type locality: Morocco, Haut Atlas, Jbel Ayachi, Mikdane.

**Distribution.** Palaearctic – Algeria, Morocco, Portugal, Spain.

***Pollenia mayeri* Jacentkovský, 1941**

*Pollenia mayeri* Jacentkovský, 1941a: 14. Type locality: Czech Republic, Brno-Bystrec, Lednice (Eisgrub).

*Polleniella distincta* Jacentkovský, 1941b: 20, 22. *Nomen nudum*.

**Distribution.** Palaearctic – Belarus, Czech Republic, Germany, Hungary, Netherlands, Poland, Romania, Slovakia, Ukraine.

***Pollenia mediterranea* Grunin, 1966**

*Pollenia mediterranea* Grunin, 1966: 899. Type locality: Italy, “Vittoria-Liguria” [possibly = Nostra Signora della Vittoria, Appennino Ligure, Liguria].

*Nitellia hermoniella* Lehrer, 2007a: 24. Type locality: Israel, Mt. Hermon, 1600–2000 m. Syn. nov.

**Distribution.** Palaearctic – Israel, Italy.

***Pollenia mesopotamica* Mawlood & Abdul-Rassoul, 2009**

*Pollenia mesopotamica* Mawlood & Abdul-Rassoul, 2009: 59. Type locality: Iraq.

**Distribution.** Palaearctic – Iraq.

***Pollenia moravica* (Jacentkovský, 1941)**

*Chaetopollenia moravica* Jacentkovský, 1941b: 21. Type locality: Czech Republic, Brno, Skolny Statek Adamov, Hády.

*Chaetopollenia pseudobisulca* Jacentkovský, 1941b: 21, 23 [key]. Type locality: Czech Republic, Brno.

**Distribution.** Palaearctic – Austria, Croatia, Czech Republic, Hungary, Poland, Romania, Slovakia, Ukraine, Yugoslavia.

***Pollenia moretonensis* Macquart, 1855**

*Pollenia moretonensis* Macquart, 1855a: 136. Type locality: Australia, Queensland, Moreton Bay.

**Distribution.** Australasian – Australia (Queensland).

***Pollenia mystica* Rognes, 1988**

*Pollenia mystica* Rognes, 1988: 322. Type locality: Georgia, Tskhratskaro, 2460 m.

**Distribution.** Palaearctic – Armenia, Georgia.

***Pollenia nigripalpis* Dear, 1986**

*Pollenia nigripalpis* Dear, 1986: 41. Type locality: New Zealand, Three Kings Islands, Great Island.

**Distribution.** Australasian – New Zealand.

***Pollenia nigripes* Malloch, 1930**

*Pollenia nigripes* Malloch, 1930: 320. Type locality: New Zealand, South Island, Westland District, Kumara.

**Distribution.** Australasian – New Zealand.

***Pollenia nigrisquama* Malloch, 1930**

*Pollenia nigrisquama* Malloch, 1930: 319. Type locality: New Zealand, South Island, Westland District, Kumara.

**Distribution.** Australasian – New Zealand.

***Pollenia nigrita* Malloch, 1936**

*Pollenia nigrita* Malloch, 1936: 22. Type locality: Australia, New South Wales, Yaouk, 1067 m.

**Distribution.** Australasian – Australia (New South Wales).

***Pollenia notialis* Dear, 1986**

*Pollenia notialis* Dear, 1986: 43. Type locality: New Zealand, Stewart Island, Table Hill, Hut Creek, 300 m.

**Distribution.** Australasian – New Zealand.

***Pollenia opalina* Dear, 1986**

*Pollenia opalina* Dear, 1986: 43. Type locality: New Zealand, South Island, Nelson District, Takaka Hill, 610 m.

**Distribution.** Australasian – New Zealand.

***Pollenia oreia* Dear, 1986**

*Pollenia oreia* Dear, 1986: 43. Type locality: New Zealand, South Island, Central Otago District, Dunstan Range, summit, 1590–1650 m.

**Distribution.** Australasian – New Zealand.

***Pollenia paragrunini* Rognes, 1988**

*Pollenia paragrunini* Rognes, 1988: 325. Type locality: Azerbaijan, Syunik, Betschenagsku Pass.

**Distribution.** Palaearctic – Armenia, Azerbaijan.

***Pollenia paupera* Rondani, 1862**

*Pollenia paupera* Rondani, 1862: 196, 200. Type locality: Malta and Gozo. [Lectotype designated by Rognes (1991c: 366).]

*Pollenia longitheca* Rognes, 1987b: 487. Type locality: Cyprus, Amathus.

**Distribution.** Palaearctic – Algeria, Cyprus, France (Corsica), Greece (Crete, Dodekanisos, mainland), Iran, Israel, Italy (mainland, Sardinia, Sicily), Malta, Turkey, Ukraine.

***Pollenia pectinata* Grunin, 1966**

*Pollenia pectinata* Grunin, 1966: 899. Type locality: Russia, Primorskiy Kray, east slope of Sikhote-Alin, valley of Sankhobe River.

**Distribution.** Palaearctic – China (Liaoning), Mongolia, Poland, Russia.

***Pollenia pediculata* Macquart, 1834**

*Pollenia pediculata* Macquart, 1834: 19(155). Type locality: France, Nord, near Lille. *Remarks*. Rognes (1991a: 234) acted as First Reviser giving *pediculata* precedence over *coerulescens*.

*Pollenia coerulescens* Macquart, 1834: 17(153) [as *cœrulescens*]. Type locality: France, Nord, near Lille.

*Pollenia obscura* Bigot, 1887: 173. Type locality: North America. Junior secondary homonym of *Musca obscura* Fabricius, 1794: 315 (= *Musca rudis* Fabricius, 1794).

*Pollenia pseudorudis* Rognes, 1985: 90. New replacement name for *P. obscura* Bigot, 1887.

**Distribution.** Afrotropical [introduced] – South Africa. Australasian [introduced] – New Zealand. Nearctic [introduced] – Canada (British Columbia, Ontario, Quebec, Saskatchewan); USA (Arkansas, California, Colorado, Delaware, Idaho, Illinois, Iowa, Kentucky, Michigan, Minnesota, Missouri, Nebraska, Nevada, New Mexico, New York, North Carolina, Ohio, Oregon, Pennsylvania, South Dakota, Utah, Virginia, Washington, Wisconsin, Wyoming). Neotropical [introduced] – Bahamas. Oriental – India, Pakistan. Palaearctic – Andorra, Armenia, Austria, Belgium, Bosnia and Herzegovina, China (Shanghai, Xinjiang, Zhejiang), Croatia, Cyprus, Czech Republic, Denmark, Finland, France (Corsica, mainland), Germany, Great Britain, Greece, Hungary, Italy, Macedonia, Netherlands, Norway, Poland, Portugal (Madeira, mainland), Romania, Russia, Saudi Arabia, Slovakia, Spain, Sweden, Switzerland, Ukraine, Yugoslavia.

***Pollenia pernix* (Hutton, 1901)**

*Gymnophania pernix* Hutton, 1901: 61. Type locality: New Zealand, South Island, Mid Canterbury District, Christchurch.

**Distribution.** Australasian – New Zealand.

***Pollenia ponti* Rognes, 1991**

*Pollenia ponti* Rognes, 1991b: 457. Type locality: Spain, Granada, 3 km NE Granada.

**Distribution.** Palaearctic – Italy (mainland, Sicily), Morocco, Portugal, Slovakia, Spain, Ukraine.

***Pollenia primaeva* Dear, 1986**

*Pollenia primaeva* Dear, 1986: 44. Type locality: New Zealand, South Island, Mid Canterbury District, Mt. Somers.

**Distribution.** Australasian – New Zealand.

***Pollenia pseudintermedia* Rognes, 1987**

*Pollenia pseudintermedia* Rognes, 1987a: 382. Type locality: Spain, Granada, Rio Guadalfeo, Orgiva.

**Distribution.** Palaearctic – Greece, Israel, Italy (Sardinia), Macedonia, Portugal, Spain.

***Pollenia pseudomelanurus* (Feng, 2004)**

*Xanthotryxus pseudomelanurus* Feng, 2004: 805. Type locality: China, Sichuan, Mt. Erlang, 3100 m.

**Distribution.** Palaearctic – China (Sichuan).

***Pollenia pulverea* Dear, 1986**

*Pollenia pulverea* Dear, 1986: 45. Type locality: New Zealand, Stewart Island, Table Hill, 425–715 m.

**Distribution.** Australasian – New Zealand.

***Pollenia rudis* (Fabricius, 1794)**

*Musca rudis* Fabricius, 1794: 314. Type locality: Germany, Schleswig-Holstein, Grömitz. [Neotype designated by Rognes (1987b: 498).]

*Musca obscura* Fabricius, 1794: 315. Type locality: Germany. [See Rognes (1987b: 496) for details.]

*Musca varia* Meigen, 1826: 66. Type locality: Germany, Nordrhein-Westfalen, probably Stolberg, near Aachen. Junior primary homonym of *Musca varia* Gmelin, 1790: 2843.

**Distribution.** Australasian [introduced] – New Zealand. Nearctic [introduced] – Bermuda; Canada (British Columbia, Ontario, Quebec, Terranova and Labrador); USA (Arizona, California, Colorado, Delaware, Georgia, Hawaii, Idaho, Illinois, Indiana, Iowa, Kentucky, Massachusetts, Michigan, Minnesota, Nevada, New Jersey, New Mexico, New York, North Carolina, Ohio, Oregon, Pennsylvania, Tennessee, Utah, Virginia, Washington, Western Virginia, Wisconsin). Oriental [introduced] – China (Guangdong), India, Nepal, Pakistan. Palaearctic – Albania, Algeria, Andorra, Austria, Belarus, Belgium, China (Shanghai) [introduced], Cyprus, Czech Republic, Denmark, Finland, France, Germany, Great Britain, Greece (Crete, mainland), Hungary, Ireland, Italy (mainland, Sardinia, Sicily), Japan (widespread), Lithuania, Morocco, Netherlands, Norway, Poland, Portugal (Azores Islands, Madeira, mainland), Romania, Russia, Saudi Arabia, Slovakia, Spain (Canary Islands, mainland), Sweden, Switzerland, Turkey, Ukraine, Uzbekistan.

***Pollenia ruficrura* Rondani, 1862**

*Pollenia ruficrura* Rondani, 1862: 196, 202. Type locality: Italy, Parma.

*Nitellia ospedaliana*: Lehrer (2007a: 21). Unavailable name; proposed without a statement that the name-bearing type will be (or is) deposited in a named collection, here listed under *Pollenia ruficrura* Rondani, 1862.

**Distribution.** Palaearctic – France (Corsica), Italy (mainland, Sardinia), Morocco.

***Pollenia rufifemorata* Rognes & Baz, 2008**

*Pollenia rufifemorata* Rognes & Baz, 2008: 391. Type locality: Spain, Sierra de Guadarrama Mts, Madrid Province, between Lozoya and Puerto de Navafria, 1400 m.

**Distribution.** Palaearctic – Spain.

***Pollenia sakulasi* (Kurahashi, 1987)**

*Dexopollenia sakulasi* Kurahashi, 1987: 68. Type locality: Papua New Guinea.

**Distribution.** Australasian – Papua New Guinea.

***Pollenia sandaraca* Dear, 1986**

*Pollenia sandaraca* Dear, 1986: 45. Type locality: New Zealand, Stewart Island, Rakeahua Valley.

**Distribution.** Australasian – New Zealand.

***Pollenia scalena* Dear, 1986**

*Pollenia scalena* Dear, 1986: 46. Type locality: New Zealand, Snares Islands, Biological Station.

**Distribution.** Australasian – New Zealand.

***Pollenia semicinerea* Villeneuve, 1911**

*Pollenia semicinerea* Villeneuve, 1911b: 51. Type locality: Syria, between Homs and Bahret Homs [Quattinah Lake]. [Lectotype designated by Rognes (1988: 333).]

*Pollenia bentalia* Lehrer, 2007c: 23. Type locality: Israel, Golan Heights, Mt. Hermon, 2000 m. Syn. nov.

**Distribution.** Palaearctic – Israel, Lebanon, Syria.

***Pollenia shaanxiensis* Fan & Wu, 1997**

*Pollenia shaanxiensis* Fan & Wu in Fan et al. 1997: 418. Type locality: China, Shaanxi, Huanglong.

**Distribution.** Palaearctic – China (Shaanxi).

***Pollenia sichuanensis* Feng, 2004**

*Pollenia sichuanensis* Feng, 2004: 804. Type locality: China, Sichuan, Mao County, 2300 m.

**Distribution.** Palaearctic – China (Sichuan).

***Pollenia similis* (Jacentkovský, 1941)**

*Dasypollenia similis* Jacentkovský, 1941b: 20. Type locality: Czech Republic, Brno, Lednice, Ráječek.

**Distribution.** Palaearctic – Albania, Austria, Czech Republic, Germany, Hungary, Poland, Slovakia, Ukraine.

***Pollenia stigi* Rognes, 1992**

*Pollenia stigi* Rognes, 1992: 104. Type locality: Morocco, Azzou-Ifrane area.

**Distribution.** Palaearctic – Morocco.

***Pollenia stolida* Malloch, 1936**

*Pollenia stolida* Malloch, 1936: 21. Type locality: Australia, New South Wales.

**Distribution.** Australasian – Australia (New South Wales).

***Pollenia tenuiforceps* Séguy, 1928**

*Pollenia tenuiforceps* Séguy, 1928: 375. Type locality: not given, probably France.

*Dasypoda angustifrons* Jacentkovský, 1941b: 8 (Czech), 58 (German). Type locality: Czech Republic, Brno, Ráječek. Syn. nov.

**Distribution.** Palaearctic – Algeria, Bosnia and Herzegovina, Czech Republic, France, Hungary, Romania, Slovakia, Slovenia, Switzerland, Ukraine.

***Pollenia townsendi* Senior-White, Aubertin & Smart, 1940**

*Pollenia townsendi* Senior-White, Aubertin & Smart, 1940: 119. Type locality: India, Himachal Pradesh.

**Distribution.** Oriental – India.

***Pollenia umbrifera* (Walker, 1861)**

*Musca umbrifera* Walker, 1861: 267. Type locality: Indonesia, Sulawesi, Tondano.

**Distribution.** Oriental – Indonesia.

***Pollenia uniseta* Dear, 1986**

*Pollenia uniseta* Dear, 1986: 46. Type locality: New Zealand, South Island, Central Otago District, Old Man Range, Hyde Rock, 1550–1650 m.

**Distribution.** Australasian – New Zealand.

***Pollenia vagabunda* (Meigen, 1826)**

*Musca vagabunda* Meigen, 1826: 72. Type locality: Germany, Nordrhein-Westfalen, probably Stolberg, near Aachen. [Lectotype designated by Rognes (1991a: 238).]

*Pollenia pulvillata* Rondani, 1862: 195, 198. Type locality: Italy, Parma.

*Pollenia hasei* Séguy, 1928: 370. Type locality: Spain, Madrid Province, Cercedilla.

*Nitellia norwegiana*: Lehrer (2007b: 5). Unavailable name; proposed without a statement that the name-bearing type will be (or is) deposited in a named collection, here listed under *Pollenia vagabunda* (Meigen, 1826).

**Distribution.** Nearctic [introduced] – Canada (British Columbia, Nova Scotia, Ontario, Prince Edward Island, Quebec); USA (Alaska, Connecticut, Maine, Massachusetts, New Hampshire, New Mexico, New York, Pennsylvania, Virginia). Oriental [introduced] – India. Palaearctic – Andorra, Austria, Belarus, Belgium, China (Shanghai) [introduced], Czech Republic, Denmark, Finland, France, Germany, Great Britain, Hungary, Italy, Latvia, Lithuania, Morocco, Netherlands, Norway, Poland, Portugal, Russia, Slovakia, Spain, Sweden, Tunisia, Ukraine.

***Pollenia venturii* Zumpt, 1956**

*Pollenia venturii* Zumpt, 1956: 79. Type locality: Italy, Florence Province, Tavarnuzze.

*Pollenia solitaria* Grunin, 1970: 480. Type locality: Russia, Krasnodar Krai: Lvovskoye, 18 km NNW Severskaya Station.

**Distribution.** Palaearctic – France, Germany, Greece, Iran, Italy (mainland, Sardinia), Netherlands, Poland, Russia.

***Pollenia vera* Jacentkovský, 1936**

*Pollenia vera* Jacentkovský, 1936: 114. Type locality: Bulgaria, Vitosha and Sliven.

Pollenia vera var. latifrons Jacentkovský, 1941b: 21. Type locality: not stated, probably Bulgaria, Vitosha and Sliven.

**Distribution.** Palaearctic – Austria, Bulgaria, Czech Republic, France, Greece, Hungary, Moldova, Poland, Romania, Slovakia, Ukraine, Yugoslavia.

***Pollenia verneri* Rognes, 1992**

*Pollenia verneri* Rognes, 1992: 98. Type locality: Spain, Jaen, 10 km W La Carolina.

**Distribution.** Palaearctic – Portugal, Spain.

***Pollenia viatica* Robineau-Desvoidy, 1830**

*Pollenia viatica* Robineau-Desvoidy, 1830: 413. Type locality: not stated, probably France, Yonne, Saint-Sauveur-en-Puisaye. [Lectotype designated by Rognes (1991b: 486).]

*Pollenia fulvicornis* Robineau-Desvoidy, 1830: 413. Type locality: not stated, probably France, Yonne, Saint-Sauveur-en-Puisaye.

*Pollenia vivida* Robineau-Desvoidy, 1830: 413. Type locality: not stated, probably France, Yonne, Saint-Sauveur-en-Puisaye.

*Pollenia pallida* Rohdendorf, 1926: 103. Type locality: Uzbekistan, Tashkent District, Ak-Tash Mts, 50 km NE Tashkent [“Ak-Tash-Gebirge, Turkestan (50 km nordöstlich von Tashkent)” as given by Rohdendorf (1928: 338)]. [Lectotype designated by Rognes (1991a: 230).]

*Pollenia luciensis* Mercier, 1930: 320. Type locality. France, Calvados, Luc-sur-Mer. [As subspecies of *Pollenia rudis* (Fabricius, 1794).]

*Pollenia carinata* Wainwright, 1940: 442. Type locality: United Kingdom, East Sussex, Lewes, Malling Hill. [As subspecies of *Pollenia rudis* (Fabricius, 1794).]

**Distribution.** Palaearctic – Armenia, Belgium, Bulgaria, Croatia, Czech Republic, Denmark, France, Germany, Great Britain, Greece, Hungary, Iran, Israel, Italy, Jordan, Kazakhstan, Kyrgyzstan, Lebanon, Malta, Moldova, Netherlands, Poland, Romania, Slovakia, Sweden, Syria, Turkey, Ukraine, Uzbekistan, West Bank, Yugoslavia.

***Pollenia viridiventris* Macquart, 1847**

*Pollenia viridiventris* Macquart, 1847: 100. Type locality: Australia, Tasmania.

**Distribution.** Australasian – Australia (Tasmania).

#### Nomen dubium and incerta sedis

*Volucella cervina* Schrank, 1803: 136. Type locality: near Ingolstadt, Germany.

#### Genus *Xanthotryxus* Aldrich, 1930

*Xanthotryxus* Aldrich, 1930: 3. Type species: *Xanthotryxus mongol* Aldrich, 1930, by original designation.

***Xanthotryxus auratus* (Séguy, 1934)**

*Pollenia aurata* Séguy, 1934: 22. Type locality: China, Xizang, Moupin.

**Distribution.** Palaearctic – China (Xizang).

***Xanthotryxus bazini* (Séguy, 1934)**

*Pollenia bazini* Séguy, 1934: 23. Type locality: China, Jiangxi, Kou-ling.

**Distribution.** Palaearctic – China (Jiangxi).

***Xanthotryxus draco* Aldrich, 1930**

*Xanthotryxus draco* Aldrich, 1930: 4. Type locality: China, Sichuan, Yellow Dragon Gorge.

**Distribution.** Palaearctic – China (Sichuan).

***Xanthotryxus ludingensis* Fan, 1992**

*Xanthotryxus ludingensis* Fan in Chen, Fan & Fang, 1992: 1204. Type locality: China, Sichuan, Luding.

**Distribution.** Palaearctic – China (Sichuan).

***Xanthotryxus melanurus* Fan, 1992**

*Xanthotryxus melanurus* Fan in Chen, Fan & Fang, 1992: 1205. Type locality: China, Sichuan, Mt. Gonggashan, Yanzigou.

**Distribution.** Palaearctic – China (Sichuan).

***Xanthotryxus mongol* Aldrich, 1930**

*Xanthotryxus mongol* Aldrich, 1930: 3. Type locality: China, Sichuan.

**Distribution.** Palaearctic – China (Sichuan), Japan (Kyushu), South Korea (Quelpart Island).

***Xanthotryxus uniapicalis* Fan, 1992**

*Xanthotryxus uniapicalis* Fan in Chen, Fan & Fang, 1992: 1206. Type locality: China, Yunnan, Weixi.

**Distribution.** Oriental – China (Yunnan).

#### Taxa tentatively assigned to Polleniidae

##### Genus *Anthracomyza* Malloch, 1928, resurrected name

*Anthracomyia* Malloch, 1927: 319. Type species: *Anthracomyia atratula* Malloch, 1927, by original designation. Junior homonym of *Anthracomyia* Rondani, 1868.

*Anthracomyza* Malloch, 1928: 360. New replacement name for *Anthracomyia* Malloch, 1927.

***Anthracomyza atratula* (Malloch, 1927)**

*Anthracomyia atratula* Malloch, 1927: 319. Type locality: Australia, New South Wales, Killara.

**Distribution.** Australasian – Australia (New South Wales).

**Remarks.** The Australian Faunal Directory lists the species as *Anthracomyia atratula* Malloch, 1927 (Elliot 2007), while it is listed as *Morinia atratula* Malloch, 1927 in the Catalogue of Life (Roskov et al. 2019). Malloch (1928: 360) proposed *Anthracomyza* as a new replacement name for his own *Anthracomyia*, correctly arguing that the latter is “preoccupied by *Anthracomyia* Rondani”. *Anthracomyza* was later listed as an unnecessary new name in the catalogue of Australasian Diptera (Kurahashi 1989), probably because Rondani (1856) originally gave the spelling *Anthracomya*, which differs by one letter and therefore does not enter into homonymy (ICZN 1999; article 56.2). However, as given by O’Hara et al. (2011), Rondani (1868) later emended his own spelling to *Anthracomyia*, and although this is now recognised as an unjustified emendation, it is an available name with separate authorship and therefore preoccupies *Anthracomyia* of Malloch (1927).

We here maintain *Anthracomyza* as a valid, monotypic genus; however, a careful examination of male and female terminalia is necessary to ascertain whether *Anthracomyza* belongs to Polleniidae.

##### Genus *Nesodexia* Villeneuve, 1911

*Nesodexia* Villeneuve, 1911a: 123. Type species: *Nesodexia corsicana* Villeneuve, 1911, by monotypy.

***Nesodexia corsicana* Villeneuve, 1911**

*Nesodexia corsicana* Villeneuve, 1911a: 123. Type locality: France, Corsica, Ajaccio, Campo d’Oro.

**Distribution.** Palaearctic – France (Corsica).

**Remarks.** According to Rognes (1991a)*Nesodexia corsicana* has the ventral and lateroventral surface of distalmost parts of acrophallus provided with scale-like spinules (Rognes 1991a), thus the species does not share a key synapomorphic character state supporting monophyly of Polleniidae. Moreover, the general habitus and, in particular, the head profile, characterised by a prominent lower facial margin, of *N. corsicana* are reminiscent of many phumosiine calliphorids. However, unlike all phumosiines, the katatergite of *Nesodexia* is bare (Rognes 1997), and more data are needed to resolve its phylogenetic position.
